# Inhibitory Effects on *Staphylococcus aureus* Sortase A by *Aesculus* sp. Extracts and Their Toxicity Evaluation

**DOI:** 10.3390/plants13101405

**Published:** 2024-05-18

**Authors:** Octavian Tudorel Olaru, George Mihai Nitulescu, Andreea Miruna Codreanu, Valentina-Andreea Calmuc, Luanne Venables, Maryna van de Venter, Cerasela Elena Gird, Cosmina-Gabriela Duta-Bratu, Georgiana Nitulescu

**Affiliations:** 1 Faculty of Pharmacy, “Carol Davila” University of Medicine and Pharmacy, Traian Vuia 6, 020956 Bucharest, Romania; octavian.olaru@umfcd.ro (O.T.O.); cerasela.gird@umfcd.ro (C.E.G.); cosmina-gabriela.bratu@drd.umfcd.ro (C.-G.D.-B.); georgiana.nitulescu@umfcd.ro (G.N.); 2 REXDAN Research Infrastructure, “Dunarea de Jos” University of Galati, George Cosbuc Street 98, 800385 Galati, Romania; miru.an@yahoo.com (A.M.C.); valentina.calmuc@ugal.ro (V.-A.C.); 3 Department of Biochemistry and Microbiology, Nelson Mandela University, P.O. Box 77000, Port Elizabeth 6031, South Africa; luanne.venables@mandela.ac.za

**Keywords:** antivirulence agents, red buckeye, bottlebrush buckeye, horse chestnut, *Daphnia magna* assay, polyphenols, MRHF cells

## Abstract

A promising strategy for combating bacterial infections involves the development of agents that disarm the virulence factors of pathogenic bacteria, thereby reducing their pathogenicity without inducing direct lethality. Sortase A, a crucial enzyme responsible for anchoring virulence factors to the cell surface of several pathogenic bacteria, has emerged as a possible target for antivirulence strategies. A series of hippocastanum species (*Aesculus pavia*, *A. parviflora*, *Aesculus x carnea*, and *A. hippocastanum*) were used to prepare ethanol- and water-based extracts for assessing their effect on *Staphylococcus aureus* sortase A. The extracts were characterized through HPLC analysis, and their polyphenols content was determined using the Folin–Ciocalteu method. The specific toxicity profile was evaluated in *Daphnia magna* using the median lethal concentration (LC_50_) and against the fibroblast MRHF cell line. The half maximal inhibitory concentration (IC_50_) values on sortase A, determined after 30 min of incubation, ranged from 82.70 to 304.31 µg/mL, with the *A. pavia* water extract exhibiting the highest inhibitory effect. The assessment of the *A. pavia* water extract on human fibroblasts revealed no significant signs of toxicity, even at a concentration of 500 µg/mL. This reduced toxicity was further validated through the *Daphnia* assay. These findings highlight the low toxicity and the potential of this extract as a promising source of future development of bacteria antivirulence solutions.

## 1. Introduction

The indiscriminate use of disinfectants and antimicrobial drugs leads to the increasing resistance of pathogens towards available treatments [1]. This is a natural phenomenon of microorganisms’ selection in response to the effect of antimicrobial drugs. In order to avoid the selection of resistant strains, new drug development strategies are focused on identifying biological mechanisms that are not critical for survival [2]. Targeting these mechanisms could diminish bacterial pathogenicity or impede its defense against attacks from the host’s immune system. [3]. Antivirulence drugs or pathoblockers have emerged as a new category of medications that disrupt the virulence factors of pathogens rather than directly killing or halting their growth, in contrast with bactericidal drugs that can inadvertently contribute to the development of resistance due to the selective pressure they impose [4,5].

The disruption of bacterial quorum sensing communication systems has emerged as a promising strategy to control virulence traits in pathogenic bacteria [6]. Bacterial exotoxins represent good targets for the development of monoclonal antibodies (mAbs), among which bezlotoxumab, tosatoxumab, and suvratoxumab are just a few examples [7,8]. Other strategies focus on interfering with the biosynthesis of functional membrane microdomains [9], the inhibition of biofilm formation, the adhesion of bacteria to surfaces or host tissues, and toxins’ neutralization [10].

Sortase A (SrtA) is an enzyme found in certain bacteria, particularly Gram-positive bacteria such as *Staphylococcus aureus*, *Streptococcus pyogenes*, and *Enterococcus faecalis*. Its polypeptide structure consist of two regions: an unstructured amino-terminal tail of non-polar fragments of the protein and a catalytic domain involved in the transpeptidation reaction [11]. Sortase A plays a crucial role in anchoring surface proteins to the cell wall [12]. These surface proteins are involved in adhesion, colonization, and immune evasion, making sortase A an attractive target for the development of antivirulence agents [13,14]. The use of natural inhibitors of sortase A is an interesting and promising approach in the field of antimicrobial research, promoting sustainability and reducing the environmental impact associated with chemical synthesis. Plant-derived products originating from ornamental or other widespread plants have the potential to make valuable contributions to pharmaceuticals. 

Polyphenols are ubiquitous compounds in plant species, and their positive impact on human health is widely acknowledged, yet not entirely understood to date. Research on the interaction between phenolic compounds and sortase A revealed that myricetin, quercetin, curcumin, and chlorogenic acid and its derivatives are effective inhibitors of sortase A in different strains of both *Staphylococcus aureus* and *Streptococcus mutans,* exhibiting dose-dependent inhibition [14,15,16,17]. 

Species from the *Aesculus* genus belong to the Sapindaceae family and are medicinal trees cultivated widely for ornamental and shade purposes. The genus comprises approximately 13 species of deciduous trees and shrubs, distributed across temperate regions worldwide, cultivated mainly for their highly ornamental value. While *A. hippocastanum* L. (horse chestnut) and *A. chinesis* Bunge (Chinese horse chestnut) are the most known species, there has been a growing interest for other species such as *A. pavia* L. (red buckeye), *A. flava* Sol. (yellow buckeye) syn. *A. octandra* Marsh., and *A. parviflora* Walt. (bottlebrush buckeye) [18,19]. The seeds of *A. hippocastanum* are the most utilized product from these species, being used to alleviate hemorrhoids and varicose veins and to treat a range of circulatory or venous issues, along with addressing post-operative edema and inflammation [20,21,22,23]. A similar chemical composition has been identified for the seeds from *A. chinesis* and *A. turbinata* Blume (Japanese horse chestnut), two oriental species with a long history in traditional medicine [24,25,26]. Although the seeds of these species have been intensively investigated for their therapeutic use in recent decades, there are several studies indicating their leaves as potential sources of biologically active constituents [27,28,29].

In the leaves of *A. hippocastanum*, *Aesculus x carnea* Zeyh., *A. glabra* Willd., and *A. parviflora*, several phenolic compounds have been identified, with the flavonoids (−)-epicatechin, quercetin, and kaempferol; proanthocyanidin derivatives; and 3-O-p-coumaroylquinic, neochlorogenic, and chlorogenic acids being the most abundant [30,31]. Escin is a valuable phytocompound from the *Aesculus* species, exhibiting a significant antibacterial effect on *Staphylococcus epidermidis* and *Staphylococcus aureus* in a concentration-dependent manner [32]. However, escin is present only in small quantities in leaves and immature fruits, thereby reducing the bactericidal potential of these extracts [33,34].

This study focuses on the determination of the inhibitory potential of leaf extracts from various *Aesculus* species against sortase A from *Staphylococcus aureus* as a promising natural, cost-effective, and sustainable approach to find solutions against antibiotic resistance. 

## 2. Results

### 2.1. Extracts Preparation

The leaves from several *Aesculus* species (*A. pavia*, *A. parviflora*, *Aesculus x carnea*, and *A. hippocastanum*) were subjected to solvent extraction followed by freeze-drying. The 12 solid extracts obtained were preliminarily tested on sortase A at a concentration of 50 µg/mL, and the extracts with an inhibition over 25% were selected for analysis. The extracts were coded with two capital letters as an indication of the plant and one lowercase letter as an indication of the solvent used. The yields for the six selected extracts are presented in Table 1. 

### 2.2. Quantitative Determination of the Phenolic Compounds

The total phenolic content (TPC) in the plant extracts was determined using the Folin–Ciocalteu method. The results are presented in Table 2 as means of the gallic acid equivalent (GAE) values together with their standard deviation (SD) values and the 95% confidence interval (CI95%). Each experiment was conducted in triplicate.

The extracts from *A. pavia* presented the highest TPC values, with no statistical difference in relation to the solvent used for extraction. The smallest value was registered for the water extract from *A. parviflora*, a value almost fivefold smaller than that of the water extract from *A. pavia*.

### 2.3. Sortase Inhibition

**Figure 1:**
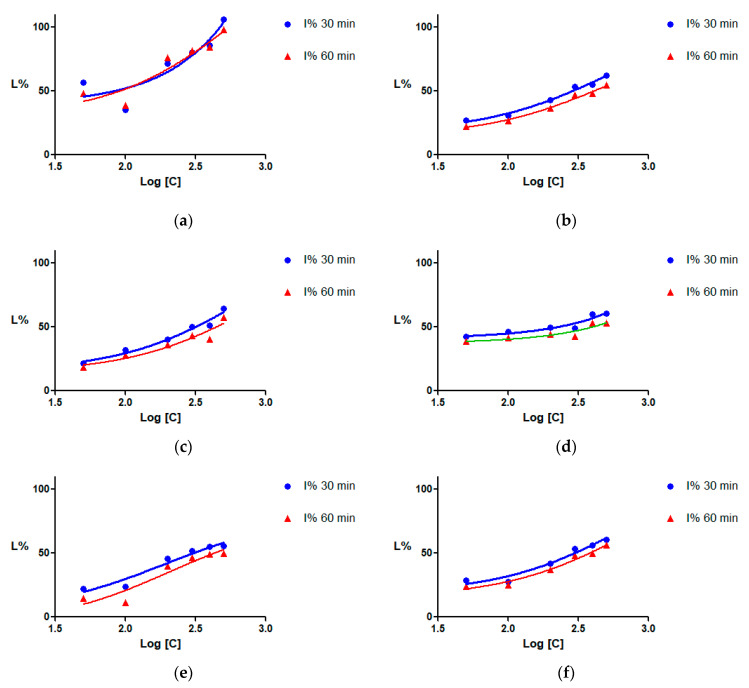
The inhibition (I%) of SrtA activity measured after 30 and 60 min of exposure to the extracts: (**a**) PVw—aqueous extract from *Aesculus pavia* leaves; (**b**) PVm—50% ethanolic extract from the leaves of *A. pavia*; (**c**) PVe—96% ethanolic extract of *A. pavia* leaves; (**d**) PRw—aqueous extract of *Aesculus parviflora* leaves; (**e**) HCe—96% ethanolic extract from leaves of *Aesculus hippocastanum*; (**f**) CRm—50% ethanolic extract from leaves of *Aesculus x carnea*.

The percentages of enzyme inhibition (I%) were plotted against the logarithm of concentrations (µg/mL), and inhibition curves were drawn using the least squares fit method (Figure 1). The I% values were dose-dependent for all the tested extracts, allowing the calculation of the half maximal inhibitory concentration (IC_50_).

For each of the four plants selected in this study, three extracts were obtained using different solvents. Each extract was preliminarily tested on sortase A at a concentration of 50 µg/mL, and the extracts with an inhibition over 25% were selected for the determination of the IC_50_ and for chemical and toxicological analysis.

All three types of extracts obtained from the *A. pavia* species (Figure 1a–c) inhibited SrtA by more than 50% at 300 µg/mL and higher concentrations. The calculated IC_50_ values are presented in Table 3. When possible, the upper and lower limits of the 95% confidence interval (CI95%) were calculated. The most potent was the aqueous extract, which presented an IC_50_ of 82.7 µg/mL after 30 min of incubation. The inhibitory capacity decreased for the extracts prepared using 50% ethanol, and even more for those prepared with 96% ethanol. For the other *Aesculus* species used (Figure 1d–f), the IC_50_ values ranged from 224.97 to 304.31 µg/mL after 30 min of incubation, and between 379.65 µg/mL and 426.95 µg/mL after 60 min.

### 2.4. UHPLC-HRMS Analysis

The quantity of phenolic compounds can vary in large limits depending on the plant environment conditions as well as on the processing methodology, including the nature of the solvent. Esculin, epigallocatechin gallate, rutin, caffeic acid, chlorogenic acid, syringic acid, and ferulic acid were identified and quantified in our extracts using a UHPLC-HRMS method. Esculin was found to be present only in PRw and HCe, while epigallocatechin gallate, rutin, chlorogenic acid, and ferulic acid were identified in all tested extracts. Syringic and caffeic acids were identified in all extracts, except in PVw and PRw for syringic acid and CRm for caffeic acid (Table 4). The full-scan total ion chromatograms for all the extracts are presented in the Supplementary Material (Figure S1), as well as those of the standard compounds (Figure S2).

The quantitative analysis of the extracts indicates significant differences between the contents of several phenolic compounds depending on the extraction solvent and on the plant species. While the contents of caffeic acid, epigallocatechin gallate, and ferulic acid were approximately equal among samples, the contents of chlorogenic acid, syringic acid, and rutin varied significantly in some extracts. The PRw extract had a content of chlorogenic acid almost 40 times higher than that of the other tested extracts, while CRm had a syringic acid content 10 times higher than that of PVm and 20 times higher than that of HCe.

### 2.5. Screening on Human Fibroblasts

**Figure 2:**
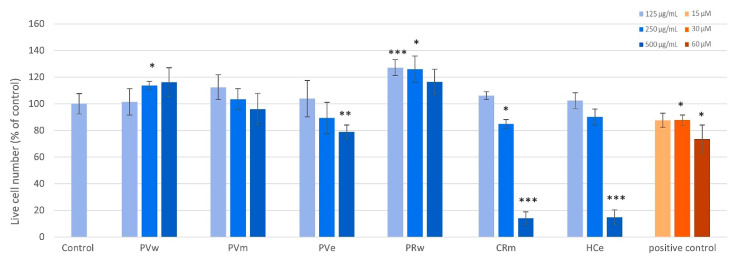
Cytotoxicity of 6 samples tested against MRHF cells. Cells were treated for 48 h. Melphalan was used as the positive control (15, 30, and 60 µM). Error bars indicate standard deviation of quadruplicate values. * *p* < 0.05; ** *p* < 0.01; *** *p* < 0.001 compared to untreated control cells.

The cytotoxicity of six samples was assessed using MRHF cells. Figure 2 shows the processed live cell data (i.e., nuclei that stained with Hoechst 33342 but not with PI) after 48 h of treatment. The samples PVw, PRw, and PVm caused a significant increase in the number of live cells at one or more of the tested concentrations and no toxicity was observed up to 500 µg/mL. CRm and HCe were non-toxic up to 250 µg/mL but significant toxicity (*p* < 0.001) was observed at 500 µg/mL. No significant changes in live cell numbers were observed with PVe at any of the concentrations compared to the control. The differences in the effects produced by the extracts can be partially explained by the varying content of polyphenols and their types.

### 2.6. Acute Toxicity Assessment Using Daphnia magna

The concentration at which lethality reaches 50%, known as the median lethal concentration (LC_50_), was determined using the interpolation method on the lethality-concentration curves. Additionally, the upper and lower bounds of the 95% confidence interval (95% CI) were calculated (Table 5). The LC_50_ values are lower for the ethanol-based extracts (50% or 96%), indicating a higher toxicity as those prepared in water.

## 3. Discussion

The sortase A inhibitors showed significant potential in addressing pathogens posing a significant risk to human health and having limited available therapeutic options, such as *Staphylococcus aureus*, *Staphylococcus epidermidis*, *Streptococcus mutans*, *Streptococcus pneumoniae*, *Streptococcus pyogenes*, *Listeria monocytogenes*, and *Bacillus anthracis* [13]. This research on natural extracts as potential inhibitors of sortase A could offer antivirulence solutions from renewable sources. This approach promotes sustainable and responsible sourcing, reducing the environmental impact associated with chemical synthesis. The ongoing search for new compounds and plant products is encouraged by the results obtained on both isolated compounds—rhodionin, orientin, morin, and quercitrin [35,36,37]—and plant materials derived from *Ocimum basilicum*, *Curcuma longa*, *Cocculus trilobus*, *Fritillaria verticillata*, or *Poncirus trifoliate* [15,38,39]. The curcuminoids from turmeric (*Curcuma longa*) are potent inhibitors of sortase A, showing potential for treating infections by inhibiting bacterial cell adhesion to fibronectin with no significant effect on bacterial growth [40]. Naturally occurring flavonols, including morin, myricetin, and quercetin, showed antivirulence properties by inhibiting sortase A and B activity [41,42].

Horse chestnut trees are widely distributed in temperate regions, and their leaves can be harvested sustainably to prepare extracts using green solvents such as water or ethanol [43,44]. Three extracts were obtained from each of the four selected plants in this study, using water, 96% ethanol, and 50% ethanol. Each extract underwent preliminary testing on sortase A at a concentration of 50 µg/mL. Extracts exhibiting an inhibition exceeding 25% were subsequently chosen for further determination of IC_50_ values, as well as for chemical and toxicological analyses. All extracts derived from *A. pavia* exhibited inhibitions overcoming the proposed threshold, whereas only one extract from each of the other species exceeded this limit, highlighting the potential of this species.

Of the extracts prepared from the leaves of the selected *Aesculus* species, the water extract from *A. pavia* (PVw) emerged as the best sortase inhibitor. The solvent used for the extraction of *A. pavia* leaves proved to be essential for the sortase A inhibitory capacity, with the IC_50_ value increasing significantly with the concentration of ethanol. Of the solvents used, water provided the best inhibitory extract also in the case of *A. parviflora* leaves. 

Even though the quantities of total polyphenols were approximately equal in PVw, PVm, and PWe (425.7 to 451.9 mgGAE/g), the HPLC results suggested that the specific polyphenol compositions were different. Natural extracts often contain a complex mixture of compounds, and identification of all the constituents is difficult but could explain the differences in activity. Considering that some coumarins can act as potent inhibitors of sortase A, the present findings may be attributed to the variation in coumarin composition among the species [33,39,45]. Although it varies slightly from one species to another, it could explain both the biological activity and the solubility [46,47]. On the other hand, the extract with the highest content of chlorogenic acid (2.47 mg/g) and rutin (12.57 mg/g), the water extract from *A. parviflora*, showed a good inhibition of sortase A.

The screening of PVw on human fibroblasts did not show toxicity, even at high concentrations of 500 µg/mL. The low toxicity was confirmed in the *Daphnia* assay where the LC_50_ was registered as 743.29 µg/mL. The other two extracts from *A. pavia* also presented low toxicity.

Even if their effect on sortase A is reduced, it is interesting to notice the high toxicity produced by high doses of CRm and HCe on both models used. This observation could be capitalized on in future studies on the leaves of *Aesculus x carnea* and *A. hippocastanum* using various other solvents and extraction methods.

## 4. Materials and Methods

### 4.1. Preparation of the Extracts

Leaves from *Aesculus* species (*A. pavia*, *A. parviflora*, *A. x carnea*, and *A. hippocastanum*) were collected from the Dimitrie Brandza Botanical Garden (Bucharest, Romania) during the blooming period and dried at 24 °C until constant weight (~one week). 

A quantity of 10 g from each material product was finely chopped and passed through a no. 4 sieve and underwent a reflux extraction process using 150 mL of solvent. The extraction process was repeated three times, and the resulting extractive solutions were combined. The ratio of solvent to the final vegetable product was maintained at 1:45. The solvents used were water, 50% (*v*/*v*) ethanol mixture with water, and 96% ethanol. The combined extractive solutions were filtered under vacuum using filter paper. The solution obtained after filtration was concentrated using a rotary evaporator (RVO001, Ingos, Prague, Czech Republic) until it reached a final volume of 50 mL. The concentrated extractive solutions were frozen and then subjected to lyophilization for 24 h at a temperature of −55 °C. The process was carried out using a ScanVac CoolSafe 55 Freeze Dryer (LaboGene, Allerød, Denmark). The dry lyophilized extracts were placed in brown glass vials, sealed tightly, and stored at room temperature in a desiccator containing calcium chloride.

### 4.2. Quantitative Determination of the Phenolic Compounds

The total phenolic content in the plant extracts was quantified using the Folin–Ciocalteu method, following a method [43,48] adapted by Olaru et al. [21]. Dilutions of each sample were prepared, and to these we added 0.6 mL of a 1/10 dilution of Folin–Ciocalteu reagent (Scharlau Co., Barcelona, Spain) and 2 mL of a 15% sodium carbonate water solution. The mixture was then incubated at 50 ± 1 °C for 15 min in a water bath. The absorbance of all samples was measured at 765 nm using a Halo DB-20-220 UV/visible spectrophotometer (Dynamica, Salzburg-Mayrwies, Germany). To establish a calibration curve, we utilized gallic acid under the same conditions. The results are reported as micrograms of gallic acid equivalents per milligram of dry weight (μg GAE/mg) of the extract. Each experiment was conducted in triplicate, and we calculated the means, standard deviation (SD), and 95% CI for each sample.

### 4.3. UHPLC-HRMS Analysis

The ultra-high performance liquid chromatography–high-resolution mass spectrometer (UHPLC-HRMS) chromatographic analysis was performed on a Vanquish Flex UHPLC System coupled with an Orbitrap Exploris 120 high-resolution mass spectrometer (Thermo Fisher Scientific, Waltham, MA, USA). Chromatographic separation was carried out on an Accucore aQ C_18_ column (100 mm × 2.1 mm, 2.6 μm) with a flow rate of 0.4 mL/min at a temperature of 40 °C. The injection volume was 5.0 μL. The mobile phase consisted of 0.05% formic acid aqueous solution (A) and 0.05% formic acid solution in methanol (B) using a gradient program (0~10 min, 5% B; 10~26 min, 30% B; 26~32.5 min, 95% B; 32.5~37 min, 5% B).

The high-resolution mass spectrometer was operated in full-scan mode (scan range 100–1000 *m*/*z*), followed by targeted MS2 scan mode. The samples were ionized with 2800 V constant current in negative ion mode with a heated electrospray ionization (H-ESI) source. The Orbitrap resolution was 120,000. The ionization source conditions were as follows: nitrogen flux was 8 units for sheath gas, 6 units for auxiliary gas, and 1 unit for sweep gas; RF lens, 50%; HCD collision energy, 30%. The vaporizer temperature was set to 320 °C and the temperature of the ion transfer tube was 300 °C [49].

Stock solutions in methanol were prepared for each of the standards used in calibration (1 mg/mL) along with a series of successive dilutions with a mixture of methanol:water:formic acid in a volume ratio of 5:95:0.05 in the range of 250–2000 ng/mL for the extracts. The solutions were stored at −20 °C. The standards and the solvents used for analysis were purchased from Sigma-Aldrich (St. Louis, MO, USA).

The analysis consisted of the detection of at least one fragment ion in comparison with the reference standards. The retention times, *m*/*z* values, and the major fragments for esculin, epigallocatechin gallate, rutin, caffeic acid, chlorogenic acid, syringic acid, and ferulic acids are presented in Table 6. Data acquisition and processing were performed using Chromeleon 7 software (Thermo Fisher Scientific, Waltham, MA, USA) with an accepted mass error of 5 ppm.

### 4.4. Inhibition of Sortase A

The extracts’ ability to inhibit SrtA activity was evaluated by measuring the fluorescence intensity arising from the breakdown of the 5-FAM/QXL^®^ substrate. This was carried out using the SensoLyte^®^ 520 Sortase A Activity Assay Kit (Anaspec, San Jose, CA, USA) [50]. To prepare the samples, the extracts were dissolved in dimethyl sulfoxide (DMSO) and then diluted with distilled water, aiming for a final DMSO concentration of 1%. The intrinsic fluorescence of the solutions was verified. Each extract was tested at six different concentrations ranging from 50 to 500 µg/mL. In accordance with the kit protocol, the assay was conducted in 96-well plates, with each well containing 10 µL of the test solution, 40 µL of the enzyme solution, and 50 µL of the substrate solution. The enzyme, the 1% DMSO solution, the substrate solution, and 4-hydroxymercuribenzoic acid (HMB) were included as control samples. The enzymatic assay was carried out at room temperature for 60 min, and the fluorescence was measured using a spectrofluorometer (SpectraMAX Gemini XS, San Jose, CA, USA) at excitation/emission wavelengths of 490 nm/520 nm. The enzyme inhibition values were calculated and plotted as a function of the logarithm of concentrations using the least squares fit method in GraphPad Prism version 5.01 software (GraphPad Software, Inc., La Jolla, CA, USA) [51,52,53].

### 4.5. Screening on Human Fibroblasts

All reagents and chemicals were purchased from Sigma-Aldrich (St. Louis, MO, USA) unless stated otherwise. Dulbecco’s Minimal Essential Medium Low Glucose (DMEM Low glucose) and PBS with and without Ca^2+^ and Mg^2+^ were purchased from Cytiva (Marlborough, MA, USA). Fetal bovine serum (FBS) and penicillin/streptomycin were purchased from Biowest (Nuaillè, France). MRHF human fibroblasts were purchased from Cellonex, South Africa. Cells were maintained in 10 cm culture dishes in complete medium (DMEM with 10% FBS and 1× penicillin/streptomycin) and incubated at 37 °C in a humidified atmosphere with 5% CO_2_.

Test extracts were reconstituted in DMSO to give a final concentration of 100 mg/mL. Samples were sonicated if solubility was a problem. Samples were stored at 4 °C until required.

MRHF cells were seeded in 96-well plates at 6000 cells/well in 100 μL aliquots and left overnight to attach. Three concentrations, namely 125, 250, and 500 µg/mL, were prepared and tested against cells and incubated for 48 h. Melphalan was used as the positive control (15, 30, and 60 µM). A vehicle control (DMSO)—untreated control—was also tested, having no effect on the cell viability. After incubation, wells were aspirated and 100 µL 5 µg/mL Hoechst was added to each well. Cells were incubated for a further 20 min. Thereafter, 10 µL PI (100 µg/mL) was added to each well, and quantification of live and dead cells was performed using an ImageXpress Micro XLS Widefield Microscope (Molecular Devices, San Jose, USA) with a 10× Plan Fluor objective and DAPI and Texas Red filter cubes. Nine image sites were acquired per well, which is representative of roughly 75% of the surface area of the well. The acquired images were analyzed using MetaXpress software (version 5.0) and the Multi-Wavelength Cell Scoring Application Module. The acquired data were transferred to an Excel spreadsheet and analyzed and processed.

### 4.6. Acute Toxicity Assessment Using Daphnia magna

The test was conducted in 4 mL 12-tissue culture wells, with each well containing 10 daphnids. The test samples were evaluated in duplicate [52]. The lethality of the organisms was recorded after 24 h, considering those that did not exhibit any movement of their appendages for 30 s as dead. All experiments were carried out in a dark environment within a plant growth chamber (Sanyo MLR-351 H, San Diego, CA, USA) maintained at a temperature of 25 ± 1 °C [52]. 

The assay was performed using six concentrations ranging from 25 to 1500 μg/mL for each extract. As for the positive control, potassium dichromate was used at concentrations ranging from 0.2 to 10 μg/mL (0.2, 0.4, 0.6, 2.0, 4.0, and 10 μg/mL, corresponding to 0.6, 1.3, 2.0, 6.8, 13.6, and 34.0 μM) based on preliminary data. The reference test with potassium dichromate [54] was performed to ensure the sensitivity of *Daphnia* and to meet the validity criterion outlined in the OECD (The Organisation for Economic Co-operation and Development) guideline 202. For this criterion, the LC_50_ of potassium dichromate at 24 h needed to fall within the range of 0.6 to 2.1 μg/mL.

## 5. Conclusions

This study underscores the potential of the *A. pavia* water extract as a promising candidate for the development of antivirulence strategies targeting *S. aureus*. The demonstrated low toxicity on human fibroblasts and validation through *D. magna* assays further emphasize the safety profile of this extract. The significant inhibitory effect on sortase A activity, coupled with the rich polyphenolic content, positions *A. pavia* as a cost-effective and sustainable source for the development of novel solutions against bacterial infections. Future research is needed to explore the extract’s efficacy in more complex biological models and assess its applicability for therapeutic applications.

## Figures and Tables

**Table 1:** The codes for the extracts from the leaves of various *Aesculus* species and the extraction yields.

Code	Plant	Solvent	Yield (% *w*/*w*)
PVw	*Aesculus pavia*	Water	22.38
PVm	*Aesculus pavia*	50% ethanol	29.98
PVe	*Aesculus pavia*	Ethanol	13.63
PRw	*Aesculus parviflora*	Water	22.75
CRm	*Aesculus x carnea*	50% ethanol	28.76
HCe	*Aesculus hippocastanum*	Ethanol	7.99

**Table 2:** The total phenolic content for the extracts determined by the Folin-–Ciocalteu method.

Code	Plant	Solvent	TPC (mgGAE/g)	CI95% (mgGAE/g)
PVw	*Aesculus pavia*	Water	425.7 ± 50.31	300.7~550.7
PVm	*Aesculus pavia*	50% ethanol	427.4 ± 40.43	286.3~327.0
PVe	*Aesculus pavia*	Ethanol	451.9 ± 66.68	286.3~617.6
PRw	*Aesculus parviflora*	Water	79.62 ± 11.27	51.62~107.6
CRm	*Aesculus x carnea*	50% ethanol	244.8 ± 5.267	231.7~257.9
HCe	*Aesculus hippocastanum*	Ethanol	270.7 ± 23.40	212.6~328.8

TPC = total phenolic content expressed as means ± standard deviation (n = 3); GAE = gallic acid equivalent; CI95% = 95% confidence interval.

**Table 3:** The inhibitory effect on SrtA after 30 and 60 min of incubation.

Code	Plant	Solvent	IC_50_ ± SD (µg/mL)		CI95% (µg/mL)	
			30 min	60 min	30 min	60 min
PVw	*Aesculus pavia*	Water	82.70 ± 2.6994	90.89 ± 2.2634	NC *	NC *~233.60
PVm	*Aesculus pavia*	50% ethanol	286.42 ± 1.8633	407.36 ± 1.8697	222.88~345.19	NC *~341.78
PVe	*Aesculus pavia*	Ethanol	315.11 ± 1.8917	441.93 ± 2.0554	220.23~412.65	258.56~NC *
PRw	*Aesculus parviflora*	Water	224.47 ± 2.1019	399.34 ± 2.3527	NC *	NC *
CRm	*Aesculus x carnea*	50% ethanol	296.80 ± 1.9330	379.65 ± 2.0387	198.92~395.02	NC *~290.03
HCe	*Aesculus hippocastanum*	Ethanol	304.31 ± 1.9312	426.95 ± 1.9197	NC *~168.70	NC *~188.19

* IC_50_ = half maximal inhibitory concentration; CI95% = 95% confidence interval; SD was calculated based on the SE of the curve; NC = not calculated.

**Table 4:** The chemical profile of the *Aesculus* sp. extracts.

Code	Concentration (μg/g Dry Extract)			Concentration (mg/g Dry Extract)			
	Esculin	Caffeic Acid	Chlorogenic Acid	Syringic Acid	Epigallocatechin Gallate	Ferulic Acid	Rutin
PVw	-	46.67	40.61	-	0.30	0.16	1.21
PVm	-	23.81	49.86	0.50	0.36	0.16	2.19
PVe	-	21.15	42.58	0.39	0.30	0.13	2.16
PRw	114.15	94.48	2472.87	-	0.30	0.12	12.57
CRm	-	-	61.09	5.52	0.37	0.17	1.52
HCe	14.17	29.24	78.46	0.27	0.35	0.15	2.39

**Table 5:** Acute toxicity of the extracts on *Daphnia magna*.

Code	Plant	Solvent	LC_50_ ± SD (µg/mL)	CI95%
PVw	*Aesculus pavia*	Water	743.29 ± 1.0735	NC *
PVm	*Aesculus pavia*	50% ethanol	371.17 ± 1.3579	NC *~741.78
PVe	*Aesculus pavia*	96% ethanol	569.44 ± 0.6281	398.35~780.00
PRw	*Aesculus parviflora*	Water	1049.81 ± 0.3042	NC *
CRm	*Aesculus x carnea*	50% ethanol	190.26 ± 0.5159	NC *~488.23
HCe	*Aesculus hippocastanum*	96% ethanol	131.15 ± 0.1957	NC *

* LC_50_ = median lethal concentration, expressed as means ± standard deviation (n = 2); SD was calculated based on the SE of the curve; CI95% = 95% confidence interval; NC = not calculated.

**Table 6:** The retention times, *m*/*z* values, and the major fragments for the tested standards.

Compound	Chemical Formula	Retention Time (min)	Adduct	*m*/*z*	Fragments
Esculin	C_15_H_16_O_9_	4.4	-H	339.0722	177.0195
Caffeic acid	C_9_H_8_O_4_	5.2	-H	179.0350	135.0450
Chlorogenic acid	C_16_H_18_O_9_	5.3	-H	353.0878	191.0562
Syringic acid	C_9_H_10_O_5_	6.5	-H	197.0455	182.0221
Epigallocatechin gallate	C_22_H_18_O_11_	6.7	-H	457.0776	169.0142
Ferulic acid	C_10_H_10_O_4_	8.8	-H	193.0506	134.0377
Rutin	C_27_H_30_O_16_	11.8	-H	609.1461	300.0272

## Data Availability

Data are contained within the article or the Supplementary Material.

## Supplementary Materials

The following supporting information can be downloaded at: https://www.mdpi.com/article/10.3390/plants13101405/s1, Figure S1: Full-scan total ion chromatogram of the new extracts; Figure S2: The chromatograms and mass spectra of the standards; Table S1: Linearity, LOD, and LOQ data.
