# Supplementary material for: Inhibitory Effects on Staphylococcus aureus Sortase A by Aesculus sp. Extracts and Their Toxicity Evaluation
Source: Plants (Basel). 2024 May 18;13(10):1405. doi: 10.3390/plants13101405 (PMC11124902; doi:10.3390/plants13101405)
Supplement: Supplementary file 1 [file plants-13-01405-s001.zip › plants-3004881-supplementary.pdf]

*Supplementary material*

# Inhibitory Effects on Staphylococcus aureus Sortase A by Aesculus Sp. Extracts and Their Toxicity Evaluation

**A**

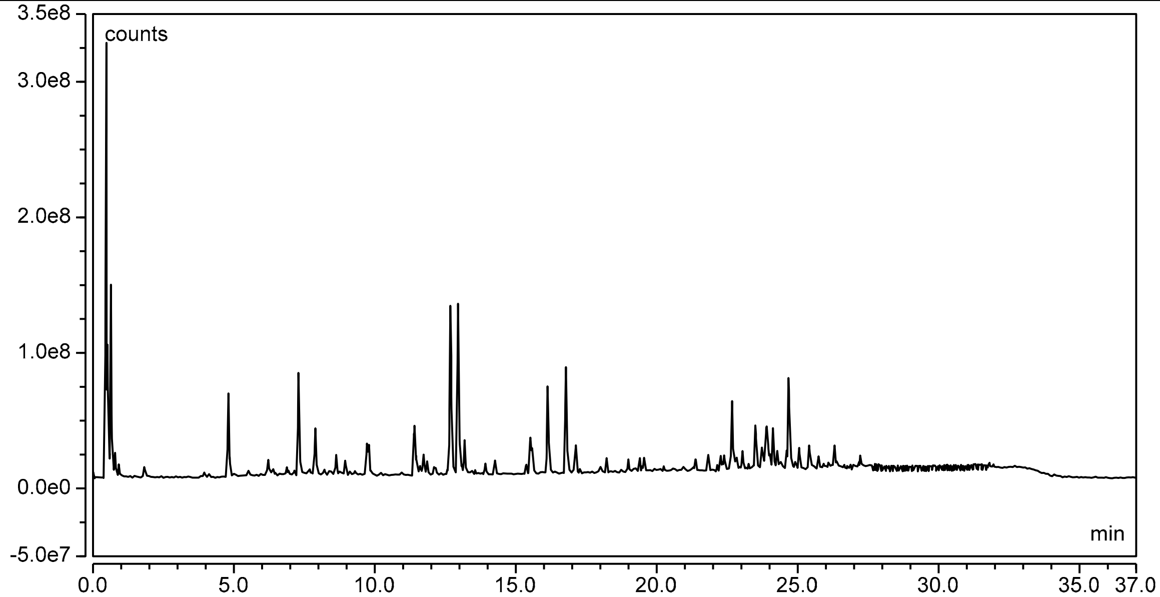


**B**

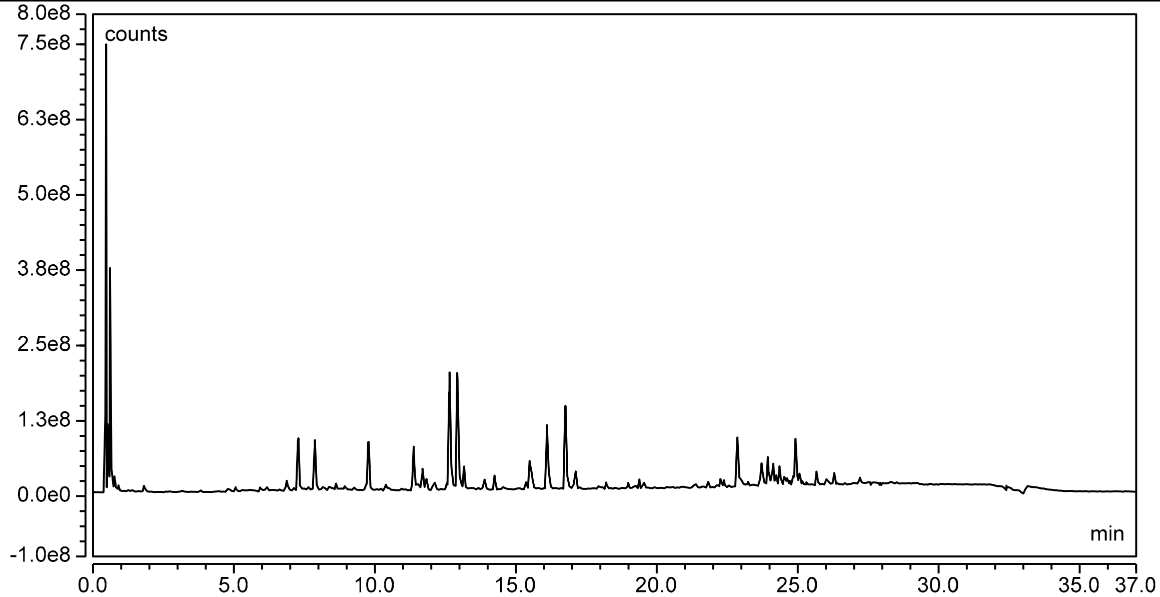

**C**

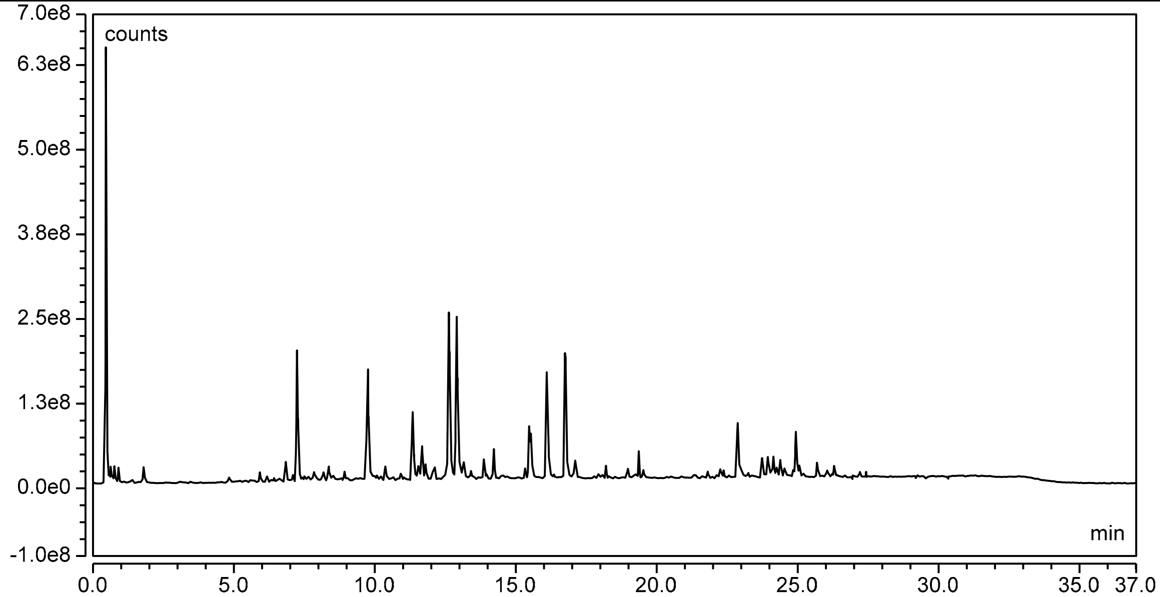
counts
min
7.0e8
6.3e8
5.0e8
3.8e8
2.5e8
1.3e8
0.0e0
-1.0e8
0.0
5.0
10.0
15.0
20.0
25.0
30.0
35.0
37.0

**D**

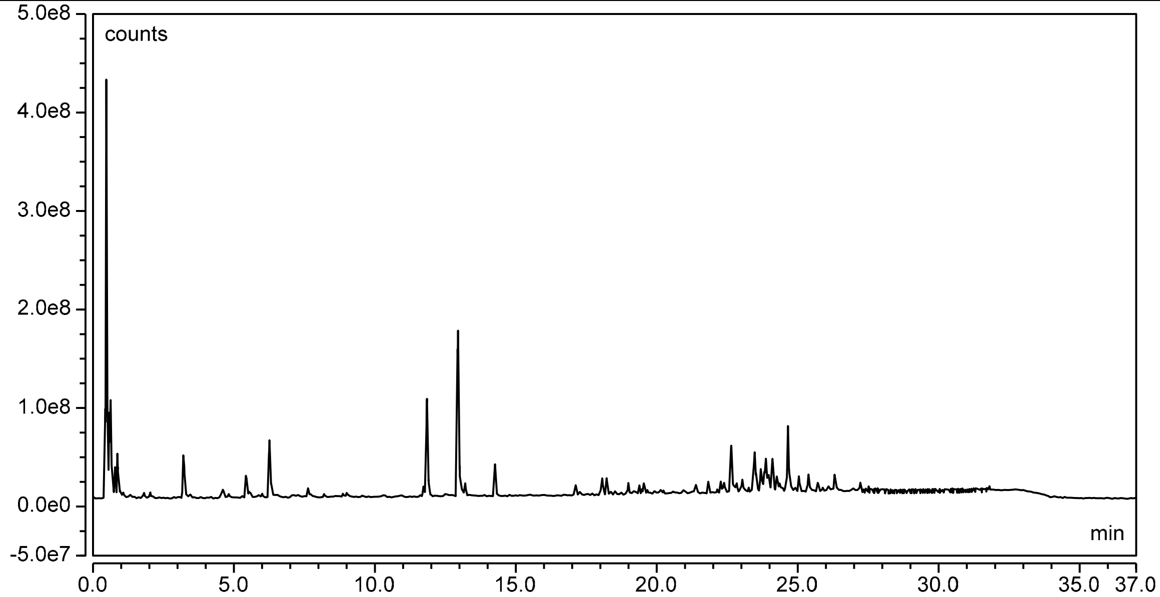
counts
min
5.0e8
4.0e8
3.0e8
2.0e8
1.0e8
0.0e0
-5.0e7
0.0
5.0
10.0
15.0
20.0
25.0
30.0
35.0
37.0

**E**

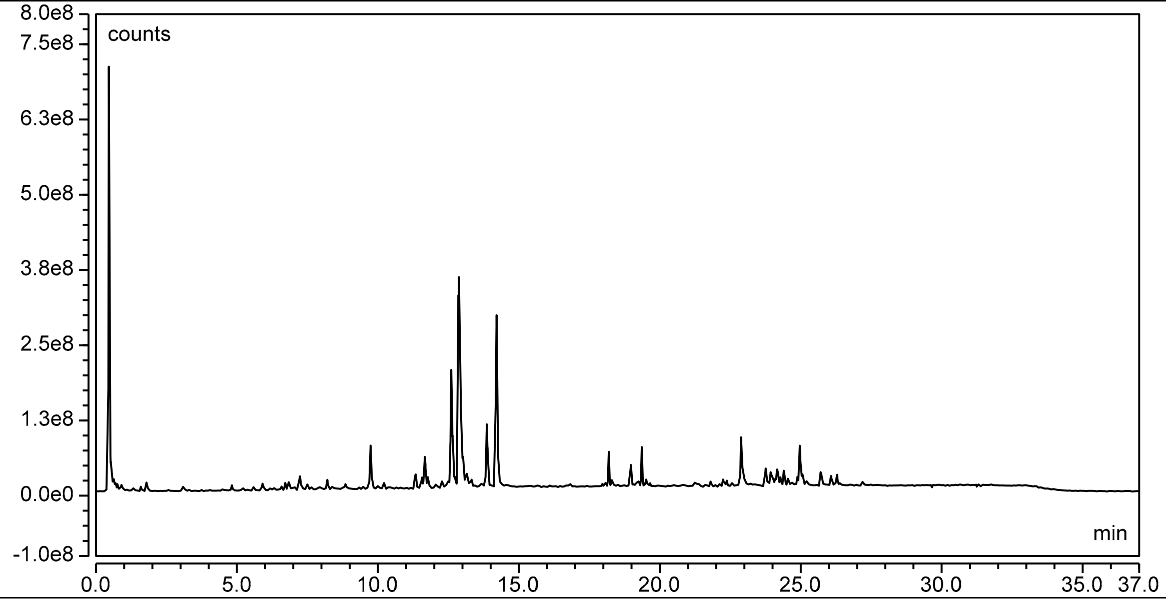
counts
min
8.0e8
7.5e8
6.3e8
5.0e8
3.8e8
2.5e8
1.3e8
0.0e0
-1.0e8
0.0
5.0
10.0
15.0
20.0
25.0
30.0
35.0
37.0

**F**

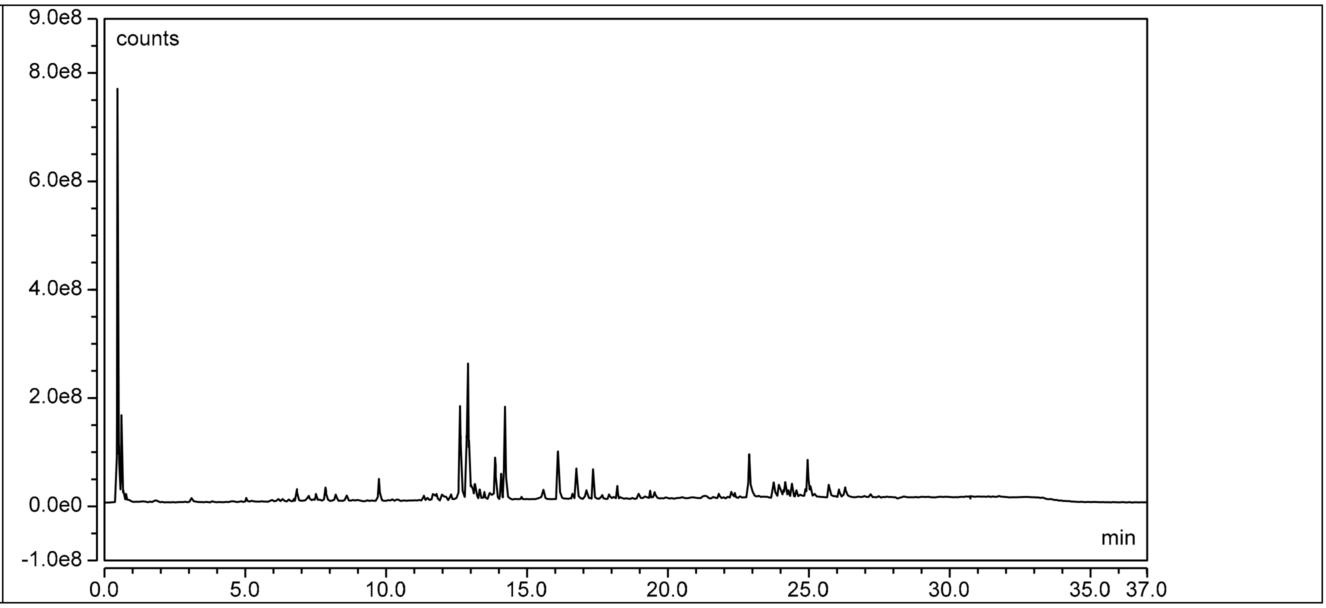


**Figure S1.** Full-scan total ion chromatogram of the new extracts: (**a**) PVw - aqueous extract from *Aesculus pavia* leaves; (**b**) PVm - ethanolic 50% extract from the leaves of *Aesculus pavia*; (**c**) PVe - ethanolic 96% extract of *Aesculus pavia* leaves; (**d**) PRw - aqueous extract of *Aesculus parviflora* leaves; (**e**) HCe - ethanolic extract 96% from leaves of *Aesculus hippocastanum*; (**f**) CRm - ethanolic 50% extract from leaves of *Aesculus x carnea*.

**A**

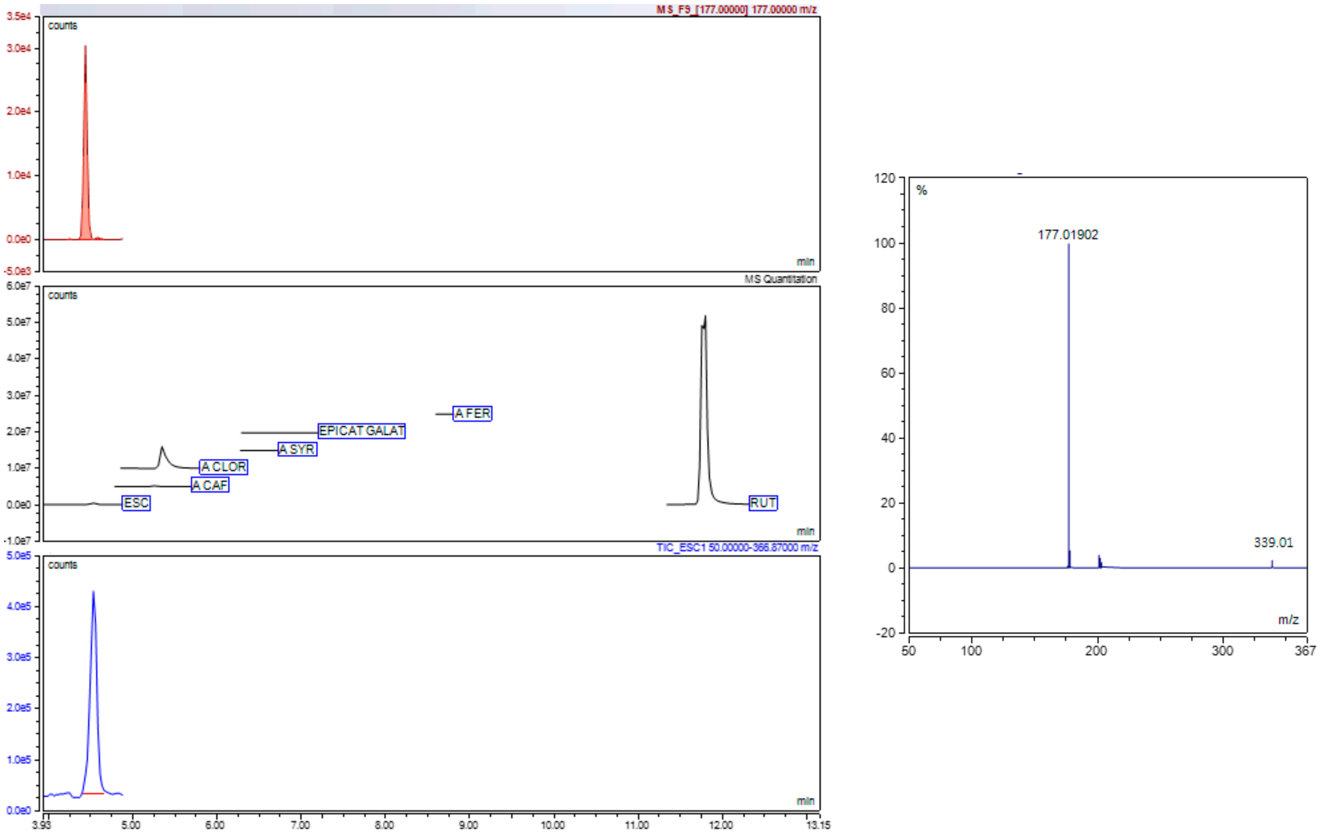

**B**

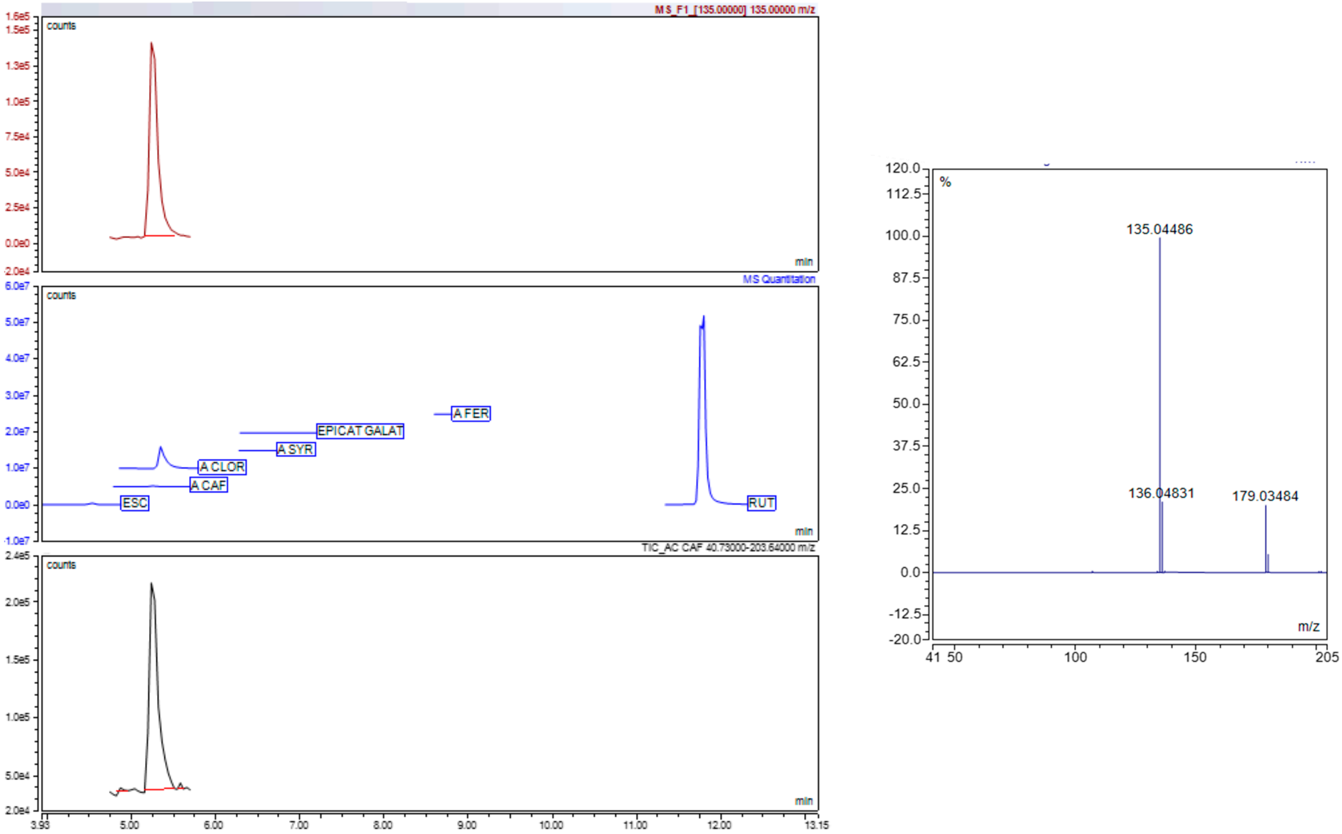
MS_F1_[135.00000] 135.00000 m/z
counts
min
MS Quantitation
ESC
A CAF
A CLOR
A SYR
EPICAT GALAT
A FER
RUT
TIC_AC CAF 40.73000-203.64000 m/z
3.93
5.00
6.00
7.00
8.00
9.00
10.00
11.00
12.00
13.15
%
135.04486
136.04831
179.03484
m/z

**C**

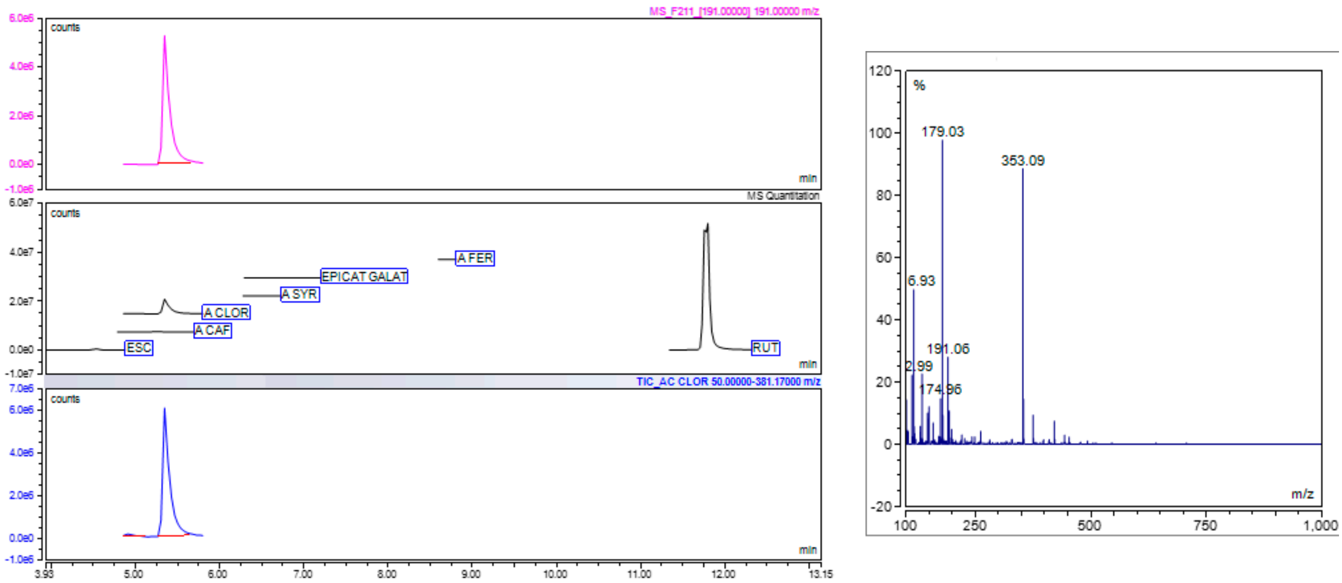
MS_F211_[191.00000] 191.00000 m/z
counts
min
MS Quantitation
ESC
A CAF
A CLOR
A SYR
EPICAT GALAT
A FER
RUT
TIC_AC CLOR 50.00000-381.17000 m/z
%
179.03
353.09
191.06
174.96
m/z

**D**

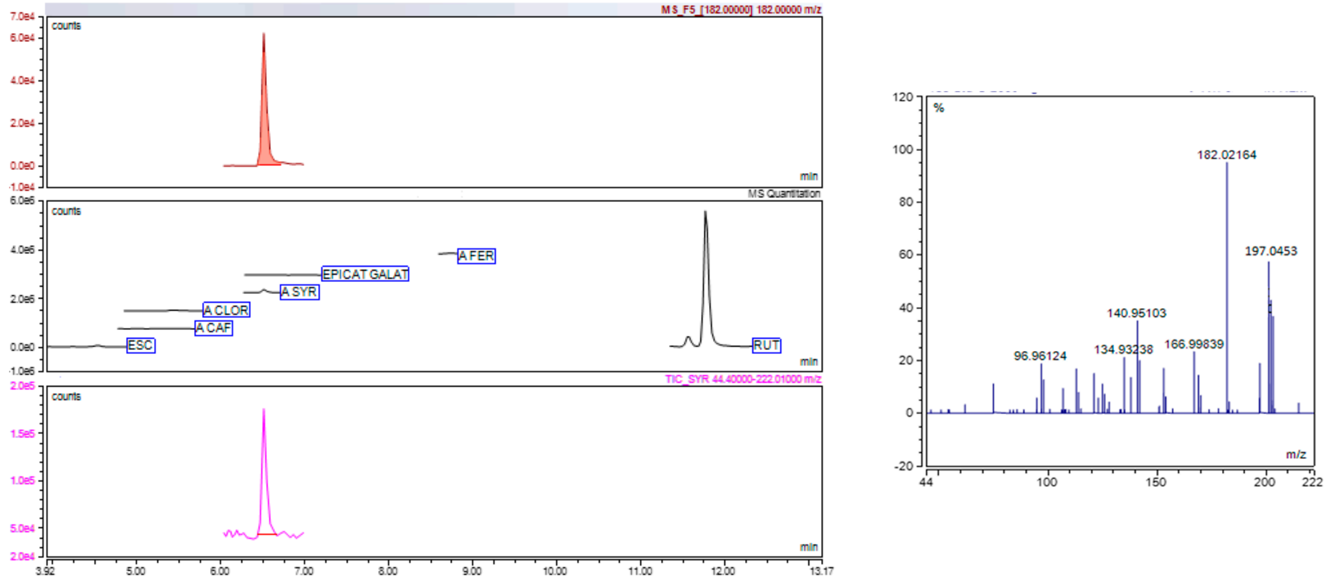
MS_F5_[182.00000] 182.00000 m/z
counts
MS Quantitation
EPICAT GALAT
A FER
A SYR
A CLOR
A CAF
ESC
RUT
TIC_SYR 44.40000-222.01000 m/z
min
%
182.02164
197.0453
140.95103
96.96124
134.93238
166.99839
m/z

**E**

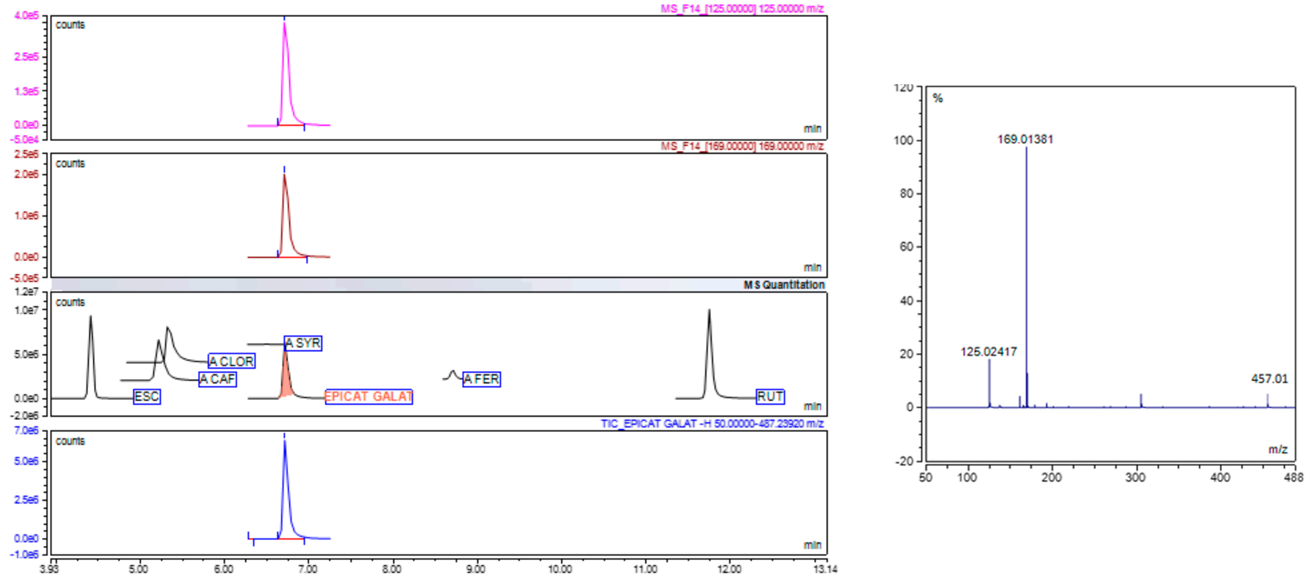
MS_F14_[125.00000] 125.00000 m/z
counts
MS_F14_[169.00000] 169.00000 m/z
MS Quantitation
A SYR
A CLOR
A CAF
ESC
EPICAT GALAT
A FER
RUT
TIC_EPICAT GALAT -H 50.00000-487.23920 m/z
min
%
169.01381
125.02417
457.01
m/z

F

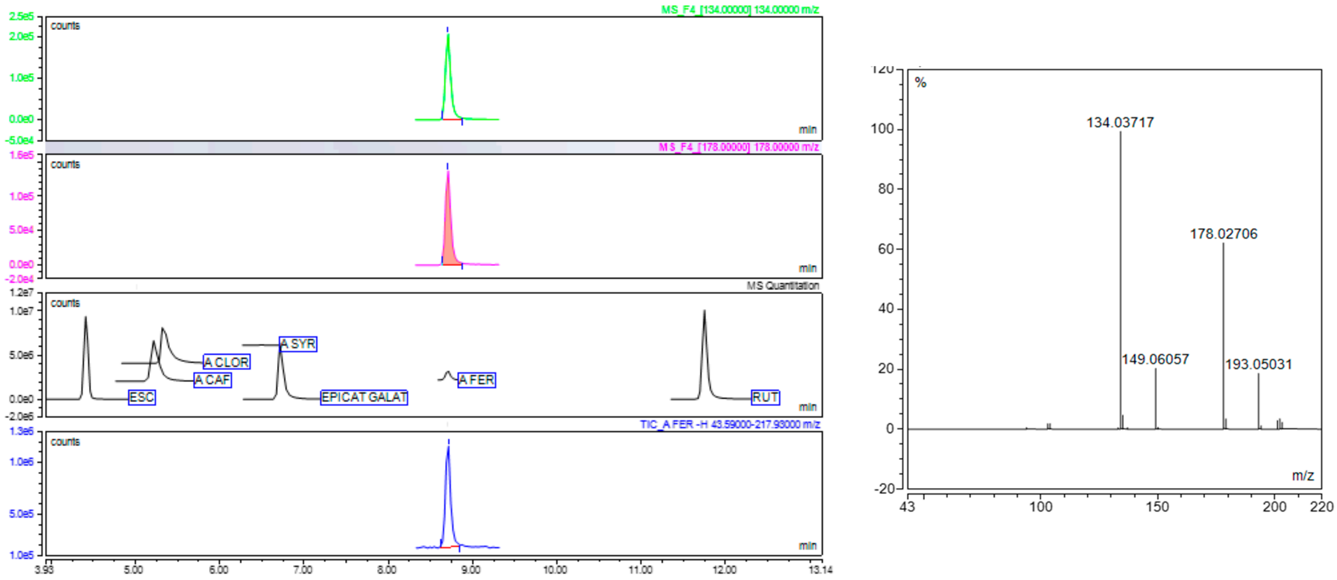


G

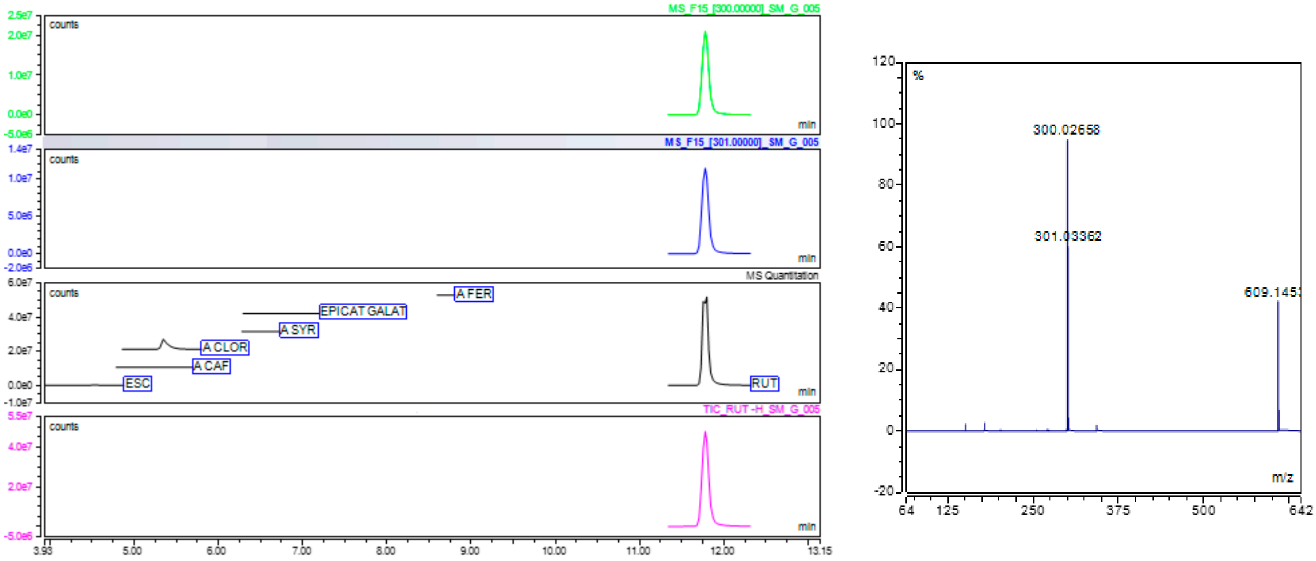


**Figure S2.** The chromatograms and mass spectra of the standards: (**a**) esculin; (**b**) caffeic acid; (**c**) chlorogenic acid; (**d**) syringic acid; (**e**) epigallocatechin gallate; (**f**) ferrulic acid; (**g**) rutin

## Analytical performance of the method

The method has provided chromatographic separation of polyphenolic compounds and detection of the target analytes via HRMS analysis within 37 minutes run time. The analytical performance of the method was expressed in terms of linearity, correlation coefficient, limit of detection (LOD) and limit of quantification (LOQ).

Linearity was evaluated by the correlation coefficient and all the target analytes have met the acceptance criteria of the calibration, respectively R2≥0.995. The correlation coefficient ranges from 0.998 to 0.999 (Table S1). A 6 level-point calibration curve was obtained for each standard, with the calibration range of 250–2000 ng/mL. Sample concentrations were calculated according to the regression parameters of the calibration curve.

The limit of detection (LOD) and the limit of quantification (LOQ) for each target compound were determined based on the calibration curve as 3.3 times and 10 times, respectively, the standard deviation of the response, divided by the slope of the calibration curve.

**Table S1. Linearity, LOD and LOQ data**

| **Compound** | **Calibration range** | **Correlation coefficient** | **LOD, ng/mL** | **LOQ, ng/mL** |
|---|---|---|---|---|
| Esculin | 250-2000 ng/mL | 0.999 | 148.25 | 449.23 |
| Caffeic acid | 250-2000 ng/mL | 0.998 | 148.64 | 450.43 |
| Chlorogenic acid | 250-2000 ng/mL | 0.999 | 169.64 | 514.05 |
| Syringic acid | 250-2000 ng/mL | 0.999 | 133.78 | 405.38 |
| Epigallocatechin gallate | 250-2000 ng/mL | 0.998 | 177.51 | 284.87 |
| Ferulic acid | 250-2000 ng/mL | 0.999 | 160.31 | 485.79 |
| Rutin | 250-2000 ng/mL | 0.999 | 121.59 | 368.47 |
